# Gut microbiota short-chain fatty acids and their impact on the host thyroid function and diseases

**DOI:** 10.3389/fendo.2023.1192216

**Published:** 2023-06-30

**Authors:** María José Mendoza-León, Ashutosh K. Mangalam, Alejandro Regaldiz, Enrique González-Madrid, Ma. Andreina Rangel-Ramírez, Oscar Álvarez-Mardonez, Omar P. Vallejos, Constanza Méndez, Susan M. Bueno, Felipe Melo-González, Yorley Duarte, Ma. Cecilia Opazo, Alexis M. Kalergis, Claudia A. Riedel

**Affiliations:** ^1^ Departamento de Ciencias Biológicas, Facultad de Ciencias de la Vida, Universidad Andrés Bello, Santiago, Chile; ^2^ Millennium Institute on Immunology and Immunotherapy, Santiago, Chile; ^3^ Department of Pathology, University of Iowa, Iowa City, IA, United States; ^4^ Facultad de Medicina Veterinaria y Agronomía, Instituto de Ciencias Naturales, Universidad de las Américas, Santiago, Chile; ^5^ Millennium Institute of Immunology and Immunotherapy, Departamento de Genética Molecular y Microbiología, Facultad de Ciencias Biológicas, Pontificia Universidad Católica de Chile, Santiago, Chile; ^6^ Center for Bioinformatics and Integrative Biology, Facultad de Ciencias de la Vida, Universidad Andrés Bello, Santiago, Chile; ^7^ Departamento de Endocrinología, Facultad de Medicina, Pontificia Universidad Católica de Chile, Santiago, Chile

**Keywords:** thyroid disorders, metabolic diseases, gut microbiota, dysbiosis, metabolism and endocrinology, Short-chain Fatty Acids (SCFAs)

## Abstract

Thyroid disorders are clinically characterized by alterations of L-3,5,3’,5’-tetraiodothyronine (T_4_), L-3,5,3’-triiodothyronine (T_3_), and/or thyroid-stimulating hormone (TSH) levels in the blood. The most frequent thyroid disorders are hypothyroidism, hyperthyroidism, and hypothyroxinemia. These conditions affect cell differentiation, function, and metabolism. It has been reported that 40% of the world’s population suffers from some type of thyroid disorder and that several factors increase susceptibility to these diseases. Among them are iodine intake, environmental contamination, smoking, certain drugs, and genetic factors. Recently, the intestinal microbiota, composed of more than trillions of microbes, has emerged as a critical player in human health, and dysbiosis has been linked to thyroid diseases. The intestinal microbiota can affect host physiology by producing metabolites derived from dietary fiber, such as short-chain fatty acids (SCFAs). SCFAs have local actions in the intestine and can affect the central nervous system and immune system. Modulation of SCFAs-producing bacteria has also been connected to metabolic diseases, such as obesity and diabetes. In this review, we discuss how alterations in the production of SCFAs due to dysbiosis in patients could be related to thyroid disorders. The studies reviewed here may be of significant interest to endocrinology researchers and medical practitioners.

## Introduction

Thyroid dysfunction is a worldwide health problem affecting an average of 20 million Americans (1, 2). The most common conditions related to thyroid dysfunction are hypothyroidism (overt and subclinical) and hyperthyroidism (overt and subclinical) (3, 4). These conditions are often associated with the development of other pathologies (5) such as depression (6) and chronic metabolic conditions like type 1 diabetes and type 2 diabetes mellitus (4, 7). Importantly, thyroid disorders are common during pregnancy (8). The prevalence of hypothyroidism during pregnancy varies between 2.5% and 11% (9). The main cause of thyroid disorders is iodine malnutrition (iodine deficiency or excess). However, genetics, smoking, alcohol consumption, infections, drugs, and gender are also important factors influencing thyroid disease development (1, 10). Maintaining a healthy lifestyle, manage thyroid and metabolic diseases, and having a balanced gut microbiome depend on appropriate nutrition (11). In this review, we discuss how having a healthy gut microbiota will facilitate the availability of essential minerals for the thyroid gland, such as iodine (12). Microbial fermentation transforms the dietary fiber into short-chain fatty acids (SCFAs) (13–15), which have beneficial effects on the host (16, 17). SCFAs enhance ATP synthesis affecting cell metabolism and supplying the gut epithelium with energy (18), and controlling the immune system (19), for example, by generating a more tolerogenic environment (20–22). Dysbiosis, or changes in the composition of the intestinal microbiota, can have an impact on the metabolism and the production of SCFAs, which can influence disease progression (23), among them are thyroid diseases. This review also provides new perspectives on SCFAs detection for improving the quality of patients’ lives.

## Thyroid hormones: an overview of normal physiological context and thyroid-related disorders

### Synthesis of thyroid hormones

**Figure 1:**
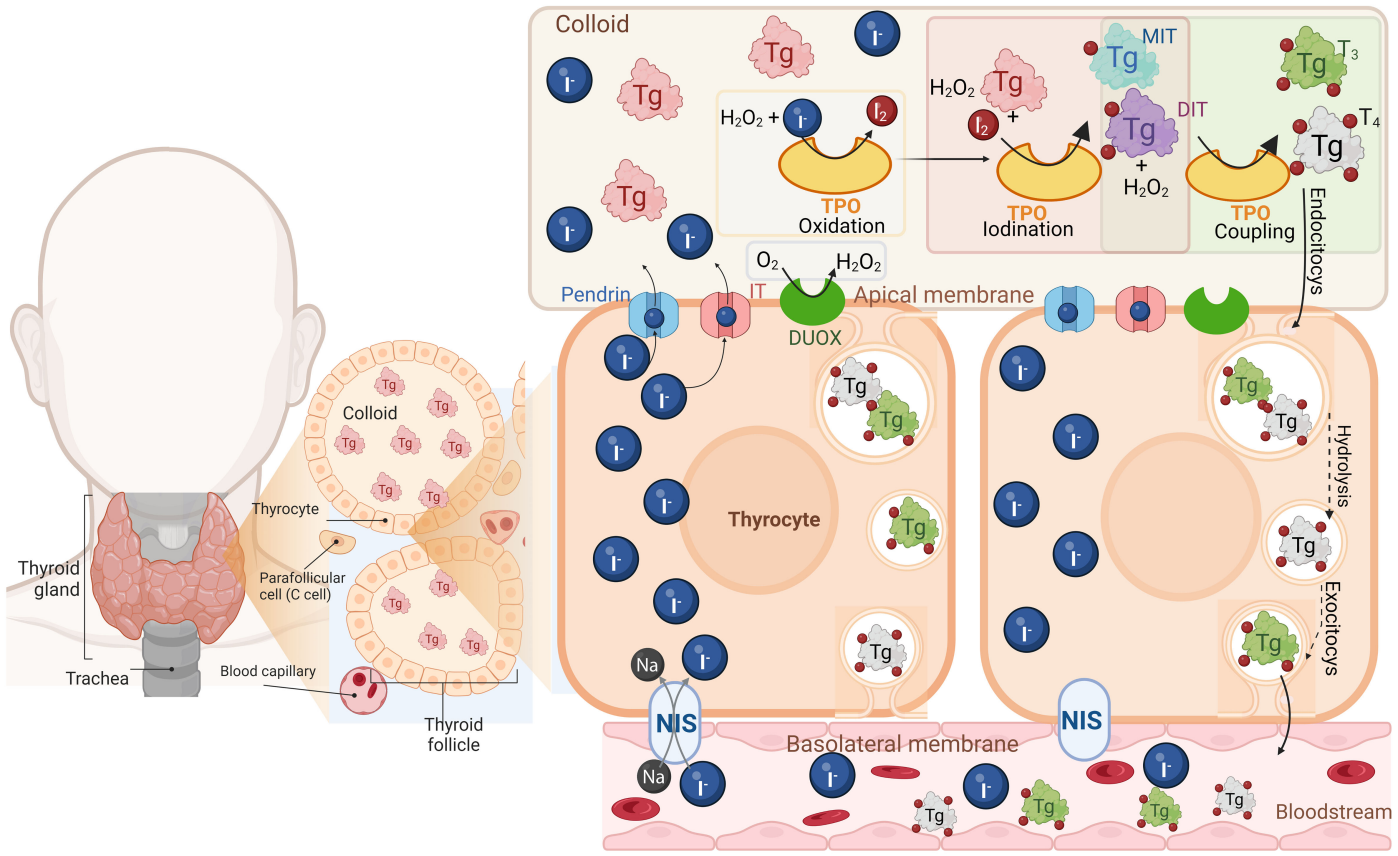
Thyroid hormone synthesis. The thyroid gland is composed of thyroid follicles which are constituted by thyrocytes, surrounding the colloid that contains thyroglobulin (Tg) the precursor of THs. Thyrocytes uptake the inorganic iodide (I^-^) through the Na^+^/I^-^ symporter (NIS), located at the basolateral membrane, diffuse and cross towards the apical membrane by the anion transporter Pendrin and the human apical iodine transporter (IT). In the apical membrane, are located the dual oxidase 1 and 2 (DUOX) enzymes which are NADPH oxidases generating hydrogen peroxide (H_2_O_2_). Then in the colloid the thyroperoxidase (TPO) catalyzes I^-^ oxidation and its covalent incorporation into a tyrosyl residue at the Tg, producing monoiodotyrosine (MIT) and 3,5-diiodotyrosine (DIT) all in the presence of H_2_O_2_. TPO also combines two DIT residues to form T_4_ and one DIT residue with one MIT residue to form T_3_ (coupling reaction). Finally, THs are endocytosed by the thyrocytes into endosomes, hydrolyzed and later released as T_4_ and T_3_ into the bloodstream. Created with Biorender.com.

Thyroid hormones (THs) L-3,5,3’,5’-tetraiodothyronine (T_4_) and L-3,5,3’-triiodothyronine (T_3_) are biological molecules that contain iodine in their structure and play crucial roles in mammal physiology like growth, neuronal development, reproduction, and cell energy metabolism (24). THs are synthesized and secreted by the thyroid gland, an endocrine gland located in the front of the neck (24, 25). Thyroid cells, called thyrocytes, are arranged into spherical structures forming the thyroid follicles, which are functional units of the thyroid gland. Each follicle surrounds a colloid mainly composed of a glycoprotein named thyroglobulin (Tg), which is the precursor of THs (24, 25). For THs synthesis, thyrocytes actively uptake inorganic iodide (I^-^) from circulation by its active transport through the Na^+^/I^-^ symporter (NIS), which is located at the basolateral side of the follicles (26). Then, I^-^ diffuses through the cytosol towards the apical membrane, and it passively crosses to the apical side of the plasma membrane through several molecules, including Pendrin and the human apical iodide transporter (IT) (27, 28). On the apical side of the plasma membrane are located the nicotinamide adenine dinucleotide phosphate (NADPH) oxidase (NOX) family of oxidoreductase enzymes, dual oxidase 1 (DUOX1) and dual oxidase 2 (DUOX2) (29–31). The primary purpose of these enzymes is to protect thyrocytes from oxidative stress as they generate hydrogen peroxide (H_2_O_2_) (30, 32). This action is not trivial because once I^-^ has been transported into the colloid, the thyroperoxidase (TPO) enzyme (in the presence of H_2_O_2_) catalyzes its oxidation (generation of I_2_) and its subsequent covalent incorporation into a tyrosyl residue at Tg (32, 33). The products of this reaction are 3-iodotyrosine or monoiodothyronine (MIT) and 3,5-diiodotyrosine or diiodotyrosine (DIT). The TPO enzyme can combine two DIT residues to form T_4_ and one DIT residue with one MIT residue to form T_3_, both reactions occur in the presence of H_2_O_2_ as oxidizing agent (32). When the thyroid gland is stimulated by TSH, the Tg is endocytosed by the thyrocytes into endosomes, which fuse with lysosomes causing the release of lysosomal enzymes that catalyze the hydrolysis of Tg residues into T_4_ and T_3_ and later will be released T_4_ and T_3_ into the bloodstream, where the majority of the THs produced in a ratio around 10:1 (T_4_/T_3_), are transported coupled to proteins such as thyroxine-binding globulin (TBG), transthyretin (TTR), and human serum albumin (HSA) (30, 33–36) (**Figure 1**).

### THs regulation and action mechanisms

**Figure 2:**
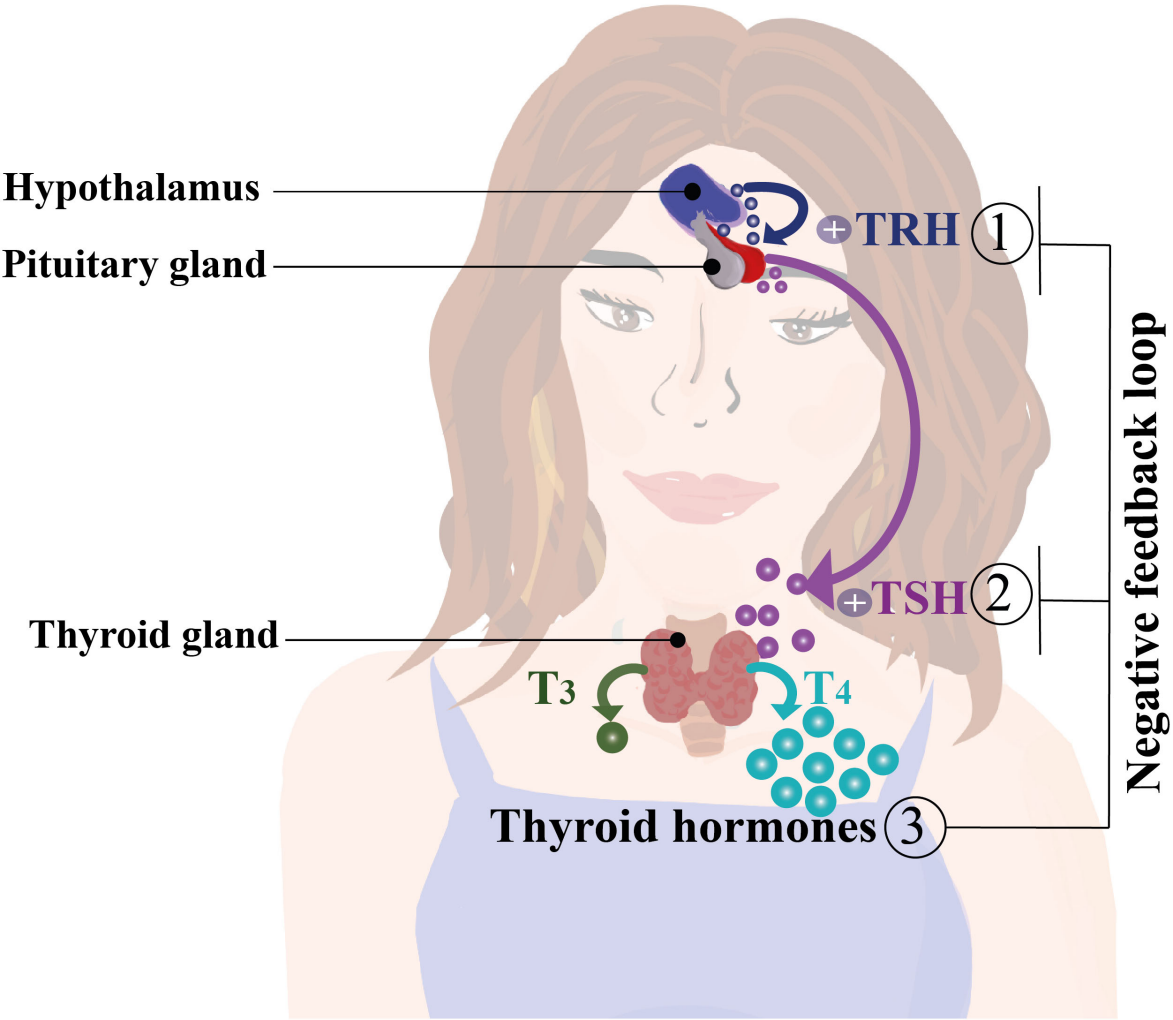
Regulation of thyroid hormone secretion and synthesis by the hypothalamus, pituitary, and thyroid gland axis (HPT axis). The hypothalamus produces and secretes thyrotropin-releasing hormone (TRH), which induces the release thyroid-stimulating hormone (TSH) by the pituitary gland. TSH stimulates the synthesis and production of T_3_ and T_4_ in the bloodstream. An increase of T_3_ and T_4_ serum levels activates a negative feedback loop over the hypothalamus and pituitary gland decreasing the production of TRH and TSH, thereby reducing the plasmatic levels of thyroid hormones (THs) (40, 42).

T_4_ comprises the majority of the THs produced and is considered the reserve hormone, whereas T_3_ is the main active biological TH (24, 37–39). THs’ production and release into the circulation are tightly regulated by the Hypothalamus-Pituitary-Thyroid (HPT) axis. Here, neurons at the hypothalamic paraventricular nucleus produce and secrete the thyrotropin-releasing hormone (TRH), which prompts the pituitary to release thyroid-stimulating hormone (TSH) (40). TSH promotes the release of T_4_ and T_3_ into the bloodstream by binding to its receptors located at the thyroid gland, stimulating the THs secretion and NIS, Tg, and TPO gene expression (40, 41) (**Figure 2**). An increase of THs in the bloodstream triggers negative feedback in the HPT axis, decreasing the production of TSH (42). T4 is converted into T3 that binds its nuclear receptor to down regulate the transcription of TSH mRNA (24, 38, 43). The conversion of T4 into T3 is mediated by deiodinases 1, 2 and 3 (D1, D2 and D3 respectively), which are selenoproteins with iodothyronine deiodinase capacity (44). In general, D1 and D2 convert T_4_ into T_3_ and reverse T_3_ (rT_3_; 3,3′,5′-triiodothyronine) into T_2_ (3,3′-diiodothyronine). D3 inactivates T_4_ by converting it into rT_3_ or T_2_ (44). Even though THs are lipophilic molecules, they are uptaken from the bloodstream by passive transport (37, 45). They can also, entry into cells by several transporters, such as monocarboxylate transporters (MCT), particularly MCT 1, 4, 8, and 10, and sodium-coupled monocarboxylate transporter 1 (SMCT1) (44). Additionally, organic anion transporters (OATP1A2 and OATP1C1) and L-type transporters 1 and 2 (LAT1/2) have also been identified as active transporters of THs (37, 45). THs can exert a genomic action *via* intracellular receptors for T_3_ (TRs), which are nuclear proteins known as TRα and TRβ, that act as transcription factors modulating gene expression (46, 47). TRs recognize specific nucleotide sequences of DNA called thyroid hormone response elements (TREs) (46, 47). These receptors are known to form complex structures with other proteins, such as the retinoic acid receptor (RXR) (46, 47). In addition to their effects on gene regulation, THs exert non-genomic mechanisms of action by binding to the transmembrane protein αVβ3 integrin, which interacts with extracellular matrix proteins (48), initiating an intracellular signaling cascade through the phospholipase C (PLC) pathway and the Cα protein, which activates MAPK proteins regulated by extracellular signals (48). Once activated, MAPK is translocated to the nucleus and phosphorylates the TRβa receptor, thereby modulating gene expression (49).

### Thyroid disorders

Thyroid disorders are conditions in which the function of the thyroid gland is impaired, and they are clinically characterized by alterations in blood THs levels (1). Hypothyroidism and hyperthyroidism are common health problems whose prevalence has been reported in 110 nations worldwide (1). In Europe, 4.94% of hypothyroidism and 1.72% of hyperthyroidism remain undiagnosed (3). A Brazilian longitudinal study showed an incidence of 1.98% for overt hypothyroidism, 0.19% for overt hyperthyroidism, 3.99% for subclinical hypothyroidism, and 0.54% for subclinical hyperthyroidism, showing a higher incidence of hypothyroidism in the country even when is a country with adequate iodine intake (10).

### Hyperthyroidism

Hyperthyroidism, clinically diagnosed by high T_3_ and T_4_ and low TSH blood levels (50), is characterized by heat sensitivity, weight loss, changes in appetite, irritability, and poor fertility (50). Among the risk factors for developing hyperthyroidism are nutritional selenium and iodine status, family history of thyroid conditions like Grave’s disease (GD), and other chronic illnesses, like pernicious anemia, primary adrenal insufficiency, and recent pregnancy, which increase the predisposition to thyroiditis (51). In America and Europe, the prevalence of overt hyperthyroidism ranges from 0.5% to 1% (3, 52). Although several therapies are available, the most effective is the use of antithyroid drugs that interfere with THs synthesis, radioiodine therapy, and surgery to eliminate a portion of the gland; however, this treatment may result in permanent hypothyroidism (53, 54). Moreover, subclinical hyperthyroidism is characterized by THs normal levels with below-normal TSH values, causing important clinical consequences like cardiovascular disease and bone loss (55).

### Hypothyroidism

Hypothyroidism is diagnosed by low blood levels of T_3_ and T_4_, with high blood TSH levels (56). Typical symptoms include fatigue, cold sensitivity, constipation, weight gain, and even more severe consequences, such as hyperlipidemia (50), most of which are consequence of a reduced metabolic rate (50, 57). One of the main causes of hypothyroidism is insufficient iodine consumption. Hashimoto’s thyroiditis (HT) is the most prevalent autoimmune thyroid condition characterized by the development of anti-thyroid antibodies, is also described as a frequent cause of hypothyroidism (58, 59). Interestingly, iodine-rich foods can serve as stressors for the thyroid gland by increasing iodine levels inside the gland and causing the production of reactive oxygen species (ROS). This results in the development of autoantibodies directed against the gland, such as TPO antibodies (TPO-Ab^+^), which is the most common trait used for the diagnosis of HT (58, 59). Globally, HT primarily affects men (approximately a 4-fold increase). However, there are variations across areas with varied economic status, which are more common in low and middle-income regions, particularly in Africa (58, 59). HT is the most common cause of subclinical hypothyroidism (60), which is characterized by normal THs levels with elevated TSH levels (4, 61). Subclinical hypothyroidism is a mild condition that could go unnoticed by the patient (60).

### Hypothyroxinemia

Hypothyroxinemia (HTX) is a frequent worldwide thyroid condition that is clinically relevant during pregnancy due to the essential role of maternal T_4_ for fetal development (62). HTX is clinically defined as low levels of blood T_4_ with normal T_3_ and TSH levels (62). Gestational HTX is 200 times more frequent than congenital hypothyroidism (63, 64). It has been reported that early in pregnancy, the maternal thyroid gland requires more iodine to supply mother and fetal THs (8). This THs demand can be a stress factor for the maternal thyroid gland, and the mother will compensate by reducing the level of T_4_ to maintain normal T_3_ levels. If this condition persists, the pregnant woman can develop hypothyroidism (8, 62, 65). Factors associated with HTX incidence are iodine deficiency, stress, smoking, and particulate matter (PM_2.5_) exposure in the air (66, 67).

### Gestational hypothyroxinemia

Gestational HTX has been linked to harmful effects on fetal development (68) due to the pivotal role of maternal T_4_ on fetal neurodevelopment (8, 62, 65). The neurodevelopmental disorders in the offspring associated with these conditions are schizophrenia, autism, bipolar disorder, and attention-deficit/hyperactivity disorder (ADHD), as well as impairments like low intelligence quotient (IQ), neurocognitive disabilities, and auditory impairments (68–74). In humans, the consequences of gestational HTX are concentrated in the CNS; however, in animal models, the consequences surpass the CNS, reaching the immune response (75, 76). The offspring gestated in HTX (HTX-offspring) have an increased predisposition to develop more severe and premature autoimmune disease in comparison to the euthyroid-gestated offspring (75). Thus, thyroid disorders are a major global health problem, and they are particularly important in pregnancy and childhood for their impact on CNS development (77). The prevalence of numerous types of thyroid disorders calls for the development of novel and less invasive treatments and additional support for diagnosis. Appropriate iodine nutrition is essential for thyroid health, and regardless of cultures, ethnicities, and/or socioeconomic status, people can be affected by iodine deficiency or excess (77, 78). However, it has been difficult to make a comparison between different countries and draw conclusions about several aspects of thyroid diseases due to differences in diagnostic thresholds, assay sensitivities, population selection, and variations in iodine intake (1).

## Nutrition and the benefits for thyroid function and gut microbiota

### Nutrition and iodine status

Many essential nutrients must be consumed in the diet; for example, iodine is an essential element for the function of all mammals’ thyroid glands and as is part of the structure of THs (79). Therefore, iodine nutrition determines thyroid function worldwide (1). Deficiency or excess of iodine in the diet can have severe consequences for fetal development, impairing central nervous system (CNS) function and body growth (80). In addition, in both infants and adults, the lack of iodine can strongly affect metabolic processes, generating hypothyroidism and goiter, whereas iodine excess can trigger thyroiditis, hyperthyroidism, and hypothyroidism (81). Consumption of foods containing iodine is the only way to obtain this micronutrient, which is present in foods such as seaweed, marine fish, and seafood (82, 83). However, historically, iodine deficiency has been supplied in many countries through the fortification of foods with massive consumption, such as table salt (78). Although this fortification succeeded in restoring normal levels of iodine intake in several countries that adhered to iodination policies. The western diet includes a low dietary fiber intake along with a high amount of ultra-processed foods containing high amounts of refined sugars, saturated fats, trans fats, salt, and alcohol (78). These components of processed foods are harmful to health and contribute to the development of diseases such as obesity, diabetes (84), metabolic syndrome (85), cardiovascular disease (86), dysbiosis (87), cancer (88), and other inflammatory diseases like inflammatory bowel disease (IBD) and asthma (89). Regarding iodine status in people who follow a western diet, some studies have indicated that normal iodine intake values decrease in this type of diet by not including key foods such as seaweed or fish, which also affects other essential micronutrients such as vitamins, selenium, iron, among others (90). On the other hand, some studies indicate that iodine intake does not decrease because of the high amounts of iodized salt used in fast and ultra-processed food preparations, but it could even deliver an excess intake of iodine, which is harmful when combined with excess fat (91). In either case, the amount of iodine ingested is not adequate, which is detrimental, especially since it is not known whether the current supplementation of table salt is beneficial. In addition, this intake is coupled with low-quality nutrients and elements (92), which not only harm the role of iodine in the body but can also affect the proper functioning of other organs besides the thyroid and cause diseases such as those previously mentioned. Therefore, more studies are required in this field because this type of diet could have more serious consequences than those currently known (93).

### Dietary fiber and their processing into beneficial products

Diet can modulate the maturation of the microbiota (94, 95) by consuming whole grains, fruits, and vegetables that provide antioxidants and fiber (96). Dietary fiber is one of the most important elements for the nutrition of microorganisms present in the intestinal microbiota, as well as for the fermentation of these compounds, by producing beneficial metabolites such as, vitamins, precursors of neurotransmitters, among others (18, 97). The metabolites produced by the intestinal microbiota can be classified as: 1) metabolites derived directly from the metabolism of dietary compounds; 2) metabolites synthesized by the host and transformed from the gut microbiota; 3) metabolites produced *de novo* by the gut microbiota (98). The metabolites modulated by dietary compounds are bile acids, branched-chain amino acids, trimethylamine, tryptophan, indole metabolites and SCFAs (98). Low fiber intake might affect intestinal barrier integrity and immunity (99)​. Fiber is an essential nutrient (11), described as a carbohydrate polymer with ten or more monomeric units that digestive enzymes cannot hydrolyze and could have a prebiotic effect (100). Dietary fibers and their different structures can elicit different microbial responses and the production of metabolites, such as SCFAs, which are one of the most studied (101, 102). SCFAs are carbohydrates with 1-6 carbon atoms. These are the final products of microbial carbohydrate fermentation by bacteria with polysaccharide-degrading enzymes, producing 90-95% of the most abundant SCFAs subtypes in the colon, including acetate (C2), propionate (C3), and butyrate (C4) (103, 104). These metabolites participate in various cellular processes and are important for maintaining intestinal and immune homeostasis (105).

## The intestinal microbiota in balance

Mothers’ health and the microbiota inherited by their offspring through gestational time, birth method, and breastfeeding influence their later adult health status (106–108). Besides, the human gut microbiota is constantly changing and maturing with its host (109). Environmental factors such as antibiotics, dietary supplements, probiotics, hygiene, and contact with pets in early life influence the offspring’s microbiota, disrupting microbial homeostasis, and increasing the risk of developing several diseases (110, 111). Subsequently, the composition, quantity, and activity of these microbial communities along the entire digestive tract can vary depending on circadian networks related to intestinal IgA (112), diet, host age, or preferential localization in the intestinal tract due to changes in pH and oxygen levels (113–115). The microbiota varies along the digestive tract, and the oral cavity is remarkably diverse and abundant (116). In mammals, the stomach harbors the lowest concentration of bacteria per gram of content (10^1^ cells/gram), followed by an increase in concentration from the duodenum to the ileum (10^3^ to 10^7^ cells/gram) and a substantial concentration in the colon (10^12^ cells/gram) (117, 118). A balanced microbiota helps in the proper development of physiological functions such as nutrient transport in the small intestine (119). Due to its anatomical location, the small intestinal microbiota has been difficult to research, despite being essential for the synthesis and assimilation of essential micronutrients, including vitamin K and B12, bile acid physiology, and digestion of carbohydrates and fats (120, 121). Most of the microbiota present in the small intestine resembles the oral cavity microbiota, with a low abundance of strict anaerobes and the most enriched bacterial species, including *Veionella, Rothia, Streptococcus, Actinomyces, Clostridium*, and *Lactobacillus* species (122, 123). The colon is inhabited by trillions of microbes that include at least 1000 distinct species that contribute enzymes that enhance human metabolism (124).

**Figure 3:**
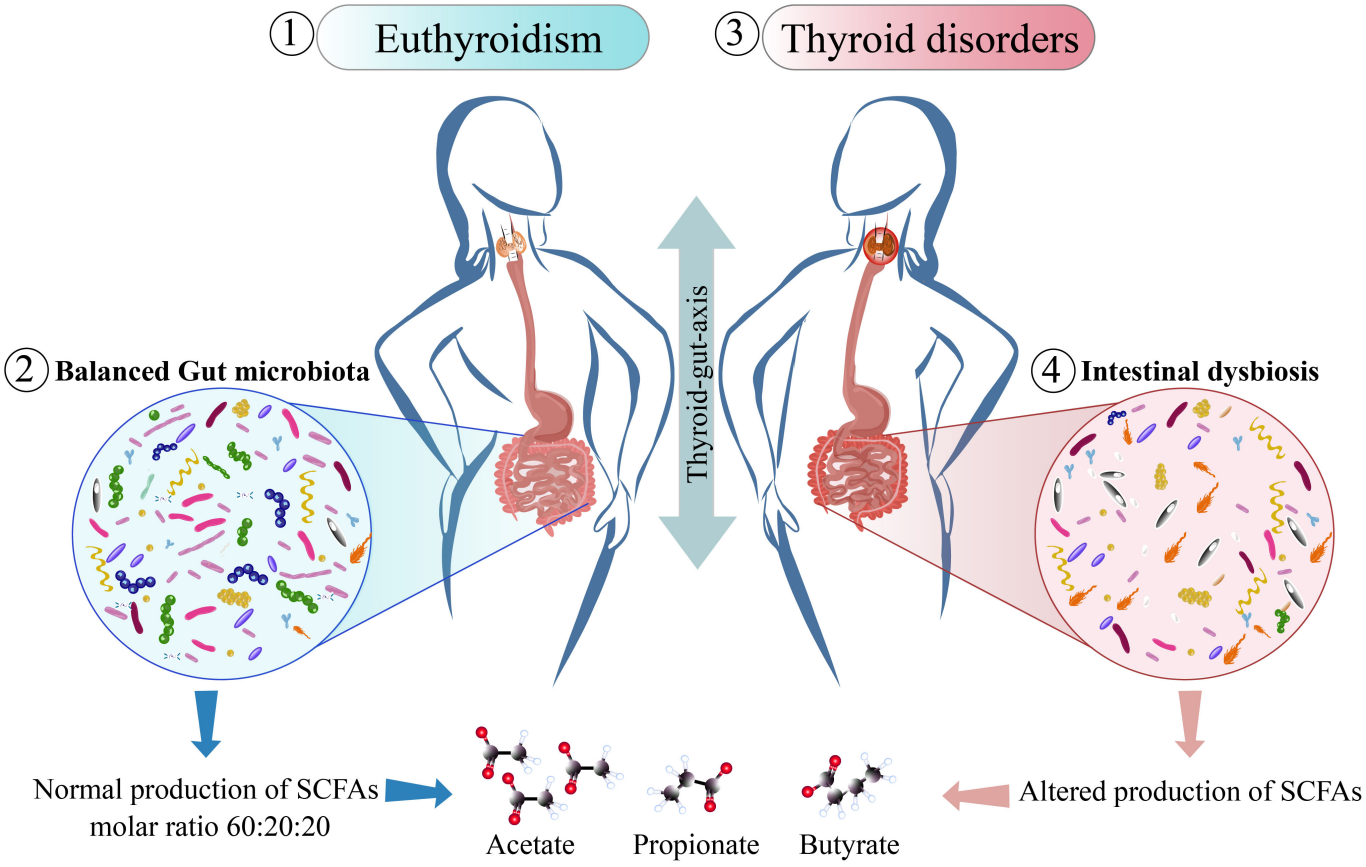
Thyroid function related to changes in gut microbiota. The figure shows how thyroid diseases influence the gut or vice-versa. Studies in individuals with normal THs levels or in euthyroidism (1), they share a gut microbiota in balance (2), with a normal production of SCFAs being acetate, propionate and butyrate produced in a molar ratio of 60:20:20 respectively (126), and when their microbiota is compared with the microbiota of patients with thyroid disorders (3), these patients shows intestinal dysbiosis with an increase in the abundance of pathogenic bacteria there is an increase in the abundance of pathogenic bacteria with a reduced abundance of commensal bacteria and reduced abundance of commensals that share functions such as SCFAs production, as it has been found in patients with Grave’s disease (127, 128).

The microbiota from the colon lives in an anaerobic enviroment coevolving with their host and can transmit genetic and metabolic characteristics to individuals (125). Within the principal metabolites produced by the this microbiota are acetate, propionate and butyrate principal SCFAs that are produced in a molar ratio of 60:20:20 respectively **(** **Figure 3** **)** (97, 126, 129). Regarding SCFAs, acetate production has been characterized in species such as *Coprococcus* sp. Strain L2-50 is dependent on the activities of butyrate kinase, acetate kinase, and butyryl-CoA: acetate-CoA transferase (130). The abundance of *Ruminococcus* and *Ruminiclostridium* genera has also been linked to fecal acetate production, where the majority is processed and absorbed in the liver, and the remaining amount of fecal acetate can be correlated with serum acetate levels (131). The pathways involved in acetate production are pyruvate *via* acetyl-CoA and the Wood-Ljungdahl pathway (132). Acetate can also be used as a substrate by butyrate-producers like *B. fibrisolvens* that have enzymes like butyril-CoA:acetate-CoA transferase; therefore, its contribution can favor butyrate production (130).

Propionate-producing bacteria harbor key enzymes for the succinate pathway or propanediol pathway (133), and the primary genera producing propionate include *Bacteroides*, *Ruminococcus, Veionella, Lactobacillus, and Propionibacterium* (131, 133, 134), using carbohydrates, organic acids, and amino acids as primary sources (133). The predominant bacteria in the colon belong to the phylum Firmicutes (135), including most members of the *Clostridia* class (135), such as *Clostridia* cluster XIV and *Faecalibacteria* (136), *Bacteroidetes*, and *Proteobacteria* (135), the majority of Gram-positive Firmicutes, which make up between 5-14 percent of the total bacteria found in healthy human feces, are colonic butyrate producers (137–139). The main producer of this metabolite are *Clostridium* cluster IV (*Faecalibacterium prausnitzii*) and *Clostridium* cluster XIVa (*Eubacterium rectale/Roseburia* spp.) (137, 138). Butyrate can be produced by the condensation of two molecules of acetyl-CoA with a later reduction to butyryl-CoA (132). Other pathways that include bacterial butyryl coenzyme A (CoA): acetate-CoA transferase and acetate kinase activities are also involved (130), using and as substrates carbohydrates, organic acids, glutamate, and lysine (133).

### Transporters and signaling of SCFAs through G-protein-coupled receptors

SCFAs are essential fecal anions absorbed in the intestinal epithelia that stimulate Na^+^-dependent fluid absorption *via* a cyclic AMP-independent process (140). Non-saturable diffusion of the undissociated acid (HSCFA) can occur through coupled electroneutral NaCl^-^ transport proteins expressed in the intestinal epithelia, enabling the exchange of Cl^−^ and HCO3^-^, as well as acetate^−^, propionate^−^, and butyrate^−^ (141).

The expression of transporters involved in the effective entry of SCFAs is critical for their local and systemic beneficial effects (142). Colonocytes preferentially obtain SCFAs *via* low-affinity H^+^ dependent monocarboxylate transporters (MCTs) or sodium-coupled monocarboxylate transporters (SMCTs) (142). SMCTs are high-affinity transporters, among them, SMCT1 (SLC5A8) and SMCT2 (SLC5A12) are expressed in the apical membrane and have electrogenic and electroneutral transport functions that regulate the luminal concentrations of SCFAs (143). Along the length of the human gut, MCT1 and MCT4 expression vary, increasing towards the distal colon (144). The transporter MCT4 (SLC16A3) present in the basolateral membrane of colonocytes, and the transporter MCT1 (SLC16A1), present in both the apical and basolateral membranes of colonocytes, are the principal transporters of SCFAs (143). Previous studies have shown that dietary fiber content may regulate the expression of MCT1, as inadequate expression levels of this transporter can be found in low-fiber diets (145). Fiber fermentation products, such as butyrate, play a critical role in regulating MCT1 gene transcription and the stability of its transcripts (146), but the expression of SMCT1 has been directly related to the modulation of the intestinal microorganisms’ producers of SCFAs (143). *In vitro* studies showed that in the presence of enteric pathogens such as *Escherichia coli*, the entry of butyrate can be altered not only by the modulation of Na^+^/H^+^ and Cl^-^/HCO3^-^ exchange but also by reducing the expression of the MCT1 transporter (147). Furthermore, since MCT1 has a basolateral distribution in enterocyte membranes, it can affect the efflux of SCFAs and monocarboxylate metabolites and the maintenance of colonic homeostasis (148, 149).

SCFAs can facilitate signaling events involving G protein-coupled receptors (GPRs), on the cell surface (150, 151). Acetate, propionate, and butyrate are among the ligands shared by some GPRs (152, 153). Within these receptors, we can find GPR109a (also known as hydroxycarboxylic acid receptor 2 or HCA2) expressed in the apical side of intestinal cells, adipose tissue, and immune cells (154), and in different tissues and cells of mammals can be found the free fatty acid receptor 2 (FFAR2; also known as GPR43) and the free fatty acid receptor 3 (FFAR3; also known as GPR41) (155, 156). The production of SCFAs by the gut microbiota can modulate immune cell responses (157). Different immune cells, such as neutrophils and leukocytes, predominate the expression of FFAR2 (158, 159) and have been described to be involved in the inhibition of inflammatory pathways (152, 153), and protective effects by diminishing the susceptibility to bacterial infections with *Klebsiella pneumoniae*, *Citrobacter rodentium*, and *Staphylococcus aureus*, but also by viruses such as respiratory syncytial and influenza viruses (159). Its protective effects can be compromised by inadequate fiber intake or a deficiency of FFAR2 (160). FFAR3 is expressed in neurons that allow neuronal links (155), blood vessel endothelial cells, and adipose tissues (158). FFAR2/3, besides regulating the immune and nervous systems, is also found in enteroendocrine cells (155). Moreover, they can activate signaling pathways related to energy metabolism, appetite control, adipogenesis, and intestinal health (156, 161), in addition to inducing signaling pathways that involve cytoprotective roles by involving nuclear erythroid 2- related factor 2 (Nrf2), contributing to the maintenance of redox homeostasis under physiological conditions, and suppressing carcinogenesis by antiproliferative properties (162).

### Effects of SCFAs in the intestinal epithelia

Intestinal fermentation of carbohydrates results in physiological concentrations of SCFAs between 10 and 100 mM in the gut lumen (163). SCFAs, such as butyrate and propionate, protect intestinal barrier integrity by influencing the expression of MUC2 mRNA levels (164). This effect is advantageous because butyrate at physiological concentrations cannot reach the epithelial stem cells in the intestinal crypts (165), since the majority is used as an energy source by differentiated intestinal epithelial cells of the villus (166, 167). It could also act as a proliferation suppressor and affect epithelial renewal, an effect not observed for propionate and acetate (165). Propionate can generate motor activity in the intestinal epithelia by enhancing the colonic flux of Cl^-^ (168). Therefore, a reduced production of SCFAs led to an increased release of lipopolysaccharide (LPS) into the bloodstream, as it has been observed in patients with hypothyroidism (169). The increase in bloodstream LPS could be related to increased thyroglobulin protein levels (170). Consequently, dysbiosis, and a decreased phylum of *Firmicutes* along with increased *Bifidobacterium* and *Lactobacillus* contribute to the inflammation in AITD (171).

### Effects of SCFAs in cellular metabolism

The intestinal microbiota has a substantial impact on energy homeostasis through SCFAs, which can act directly as the primary source of the energy produced in eukaryotic cells in the through major biochemical pathways, such as the tricarboxylic acid (TCA) cycle, oxidative phosphorylation (OXPHOS), and mitochondrial fatty acid β-oxidation (FAO) (172). FAO is a key source of energy for fatty acids metabolism based on the individual’s cellular energy requirements (173). Butyrate is the principal SCFA involved in the maintenance of energy homeostasis. When glucose is unavailable, fatty acids serve as the body’s main source of energy (174). Butyrate was found to increase FAO in keratinocytes (99) due to its ability to regulate FAO-related enzymes such as acyl-CoA dehydrogenase. In colonocytes, butyrate has a function as an energy source rather than an inhibitor of histone deacetylases (HDAC) supporting energy homeostasis and preventing autophagy (166). The mechanism of butyrate can be due to its incorporation to the TCA cycle (especially in citrate) and OXPHOS, producing Acetyl-coenzyme A (acetyl-CoA) to generate ATP (99, 165, 175). Butyrate can produce metabolic reprogramming and affect the behavior of immune cells. In macrophages, butyrate increases OXPHOS for alternative cell activation and anti-inflammatory effects (176). Moreover, it induces the peripheral inducible T regulatory cells (iTreg) by the transformation of butyrate by acyl-CoA synthetase short-chain family member 2 (ACSS2) producing butyryl-CoA (BCoA), which plays a critical role in enhancing FAO stress (177). Additionally, butyrate has been linked to the gut-brain neuronal circuit, improving plasma lipid metabolism and insulin sensitivity, promoting fat oxidation and activating brown adipose tissue (BAT) by inhibiting orexigenic neuron NPY activity in the hypothalamus (178). White adipocytes also take part in several immunological and metabolic processes (179). These cells express SCFA receptors, and it has been suggested that adipocyte metabolism of adipocytes may be influenced by these products (156, 158). *In vitro* studies have shown that SCFAs may affect glucose metabolism by increasing glucose uptake, fatty acid synthesis (180). It has been reported that, oral administration of propionate and butyrate can improve glucose and insulin tolerance in rats (181, 182). Additionally, several studies conducted in mice orally fed with acetate or butyrate showed a reducion in body fat content due to improved energy expenditure and fat oxidation (181, 183). The evidence available in the literature strongly suggests that SCFAs play a beneficial role in host metabolism; however, there are not enough *in vivo* studies conducted in humans to support the physiological effects of SCFAs. More studies are needed to understand the impact of SCFAs on glucose and fat metabolism and insulin sensitivity, particularly in adipose tissue.

### Regulation of immune function by SCFAs

The majority of immune cells, including B cells, CD4 T cells, CD8 T cells, macrophages, dendritic cells (DCs), innate lymphoid cells (ILCs), and polymorphonuclear leukocytes, express one or more of the GPRs. Thus, SCFAs can bind to these receptors on immune cells to modulate their function. CD4 regulatory T cells (Tregs) are a subset of CD4 T cells that express FoxP3 and/or produce IL-10 and are responsible for the regulation of proinflammatory IFN-ϒ producing Thelper-1 (Th1) and IL-17 producing Th17 cells. The intestine has a large Treg population that maintains homeostasis at the mucosal surfaces, where intestinal bacteria and metabolites play an important role in the generation or maintenance of this Treg population. SCFAs are one of the major bacterial products linked to the expansion and suppressive function of Treg (184, 185). For example, SCFAs from chloroform-resistant fecal bacteria were established as the main bacterial metabolite responsible for the induction of Tregs and suppression of inflammation in a murine model of colitis (186, 187).

Although the precise mechanism through which SCFAs influence Tregs is unknown, SCFAs are thought to enhance the suppressive function of Treg cells (184, 185). Administration of dietary fibers, SCFAs, and gut bacteria with SCFAs-producing ability has been shown to induce Treg cells, and it has been implicated in this by histone deacetylation function of SCFA. HDACs can modify chromatin structure and control genes by removing acetyl groups from specific lysine residues in histone and non-histone proteins (188), which is linked to the regulation of the expression of *FoxP3* and *IL-10 genes* during steady state. Thus, the regulatory vs. inflammatory effects of SCFAs might be context dependent and in steady state, SCFAs will promote the expansion of regulatory cells such as Tregs; however, during chronic inflammatory stages or at super high levels, they might promote a proinflammatory Th1/Th17 response. SCFAs can influence the functions of APCs by modulating their cytokine production and antigen presentation function through GPR and HDAC activation. Butyrate can affect differentiation, maturation, and function of DCs (189–191).

SCFAs can also regulate Treg expansion and function indirectly through modulation of antigen presenting cells (APCs), such as DCs and macrophages. Consequently, during the steady state, SCFAs can boost Treg expansion and function. However, SCFAs have also been shown to induce pro-inflammatory Th1 and Th17 cells during an active immune response. Park et al, showed that SCFAs can induce both effectors as well as Tregs through histone modification (192, 193).

Butyrate, can signal through the receptor for SCFAs GPR109a in macrophages promoting the expansion of Tregs by DCs (194). Similarly, intestinal macrophages treated with butyrate showed reduced levels of LPS induced nitric oxide, IL-6, and IL-12 (195), and can also induce IL-10 producing regulatory B cells, especially IL-10^+^ IgM^+^ CD138^high^ plasma cells (196), both *ex-vivo* and *in-vivo*. SCFAs, particularly butyrate and propionate, can enhance class-switch DNA recombination at low doses but decrease at higher doses, suggesting a dual dose-dependent effect of SCFAs on B cell class switch (197). These studies highlight that SCFAs, especially butyrate can have significant effects on B cell function and antibody production. Besides these immune subsets, SCFAs can also influence ILCs by increasing ILC-3 activity while downregulating ILC-2 activity, which has been discussed in detail elsewhere (19). Thus, SCFAs can regulate the generation and function of most of the immune cell population; however, further studies are required to understand the precise mechanism through which SCFAs regulate different immune subsets.

## Dysbiosis, a common finding in metabolic and thyroid disorders

According to (198), dysbiosis is an imbalance between commensal and pathogenic microorganisms in the microbiome. Dysbiosis can be divided into three main categories: first as a bloom of pathobionts or low relative abundance of bacteria caused by abnormalities in the intestinal ecosystem; second as the loss of commensals due to microbial death or deduced bacterial proliferation; and third as a reduction of alpha-diversity or richness of species within a site associated with different pathologies (199).

### Dysbiosis is linked to metabolic conditions

Changes in the composition and balance of the main intestinal microorganisms are associated with an increased risk of obesity (200), IBD (201), and the pathogenesis of irritable bowel syndrome (202). Recent studies have shown a connection between the gut-thyroid axis due to the role of gut microbiota in thyroidal metabolism (203). Patients treated for Hypo or Hyperthyroidism share gut microbiota traits that are consistently associated with diseases such as depression, migraine, gout, type 2 diabetes, cardiac diseases, food allergies, constipation, and IBD (204).

The intestinal microbiota is not the only one that can be affected; in a study performed on patients with high TSH levels and insulin resistance, their oral microbiome showed more bacterial richness and prevalence of microorganisms from the genera *Granulicatella*, *Treponema*, and *Streptobacillus*, with functional profiles related to carbohydrate metabolism, nucleotide, amino acid and energy metabolism (205). Thyroid hormones play a key role in several signaling pathways (40), and are implicated in regulating body temperature by stimulating fatty acid β-oxidation, mitochondrial respiration, biogenesis, and autophagy activity in brown adipose tissue (BAT) and white adipose tissue (WAT) (206, 207), may influence free fatty acid (FFA) and its uptake in the liver (208). More specifically, FT_4_ and TSH have also been implicated in weight regulation (209), as it has been observed how HDL cholesterol level and waist circumference in patients with metabolic syndrome have been linked to thyroid function (210). Thyroid dysfunction is also prevalent in obese subjects and is an important risk factor for cardiovascular disease and diabetes (211). In a study, it was shown that among thyroid disorders, 27.6% of patients had type 2 diabetes (T2D) and 38.7% had type 1 diabetes (T1D) (4). A cross-sectional study reported an increased risk of overt hypothyroidism and TPOAb positivity among adult Iranian overweight and obese individuals (212).

Dietary fiber intake is associated with a reduced risk of non-communicable chronic diseases (213), and the American Diabetes Association (ADA) recommendations have been associated with significantly lower blood pressure in adult patients with T1D (214), whose pathogenesis involves the action of autoreactive T cells through islet ß-cells, which can be regulated by acetate (150). In the cases of T2D and obesity, both conditions show complex alterations in SCFAs levels, leading to diverse outcomes. For instance, increased butyrate production improves insulin sensitivity, whereas aberrant propionate production or absorption increases the risk of T2D (215). Demonstrating that SCFAs balance is also necessary. On the other hand, obesity is a disease with increasing prevalence, and it is characterized by an excessive accumulation of white adipose tissue that leads to negative effects on health (179, 216). It has been described that both, the composition of the gut microbiota and the availability of THs play a key role in the regulation of metabolism and whole-body energy expenditure (18, 217). Therefore, it is believed that gut microbiota products, particularly SCFAs, together with THs may modulate the progression of diseases like obesity through modifications of the host’s metabolism. The majority of WAT is made up of white adipocytes, a type of cell that is particularly adept at storing energy, primarily in the form of triglycerides. White adipocytes modulate several immunological and metabolic processes through the secretion of various hormones, cytokines, and adipokines (179). It has been shown that the SCFA receptors GPR41 and GPR43, both of which may regulate cell metabolism and function, are expressed by white adipocytes (156, 158). In line with this, it has been described that SCFAs mediate energy balance through the adipose tissue by increasing energy expenditure and fat oxidation (180, 218). Furthermore, T_3_ regulates the whole machinery responsible for the balance between lipogenesis and lipolysis in WAT (219). Nevertheless, the link between SCFAs, thyroid function, and obesity remains under discussion. It has been described that the microbiota from animals fed a western diet (high fat and high sugar diet) together with sedentarism result in a predominance of Firmicutes over the phyla accompanied by a lower degree of diversity when compared to a low fat, high polysaccharide diet (220). Obesity-induced changes in the microbiota enrichment may thus be suggested as an additional factor to explain the reported correlation between thyroid diseases and obesity.

Once established that a patient suffers from type 1 or 2 diabetes, prediabetes and gestational diabetes mellitus, a fundamental approach in the therapy is a change in lifestyle, and the ADA recommends not only physical activity, smoking cessation, and psychosocial care, but also medical nutrition therapy, emphasizing nutrient-dense carbohydrate sources high in fiber (221). With recommended fiber intake of 14 g per 1,000 kcal (222). It has been described that insulin secretion by pancreatic islets was related to increased degradation of GABA modulated by 4-aminobutanoate degradation V microbial pathway, the pathway of 4-aminobutanoate (GABA) degradation pathway have been related to the production of butyrate and acetate, associated with the abundance of species like *Eubacterium rectale* and *Roseburia intestinalis* (215). In addition, human islet ß-cell function can be beneficially changed by propionate as an acute effect to protect these cells from apoptotic stimuli (105). A study conducted on monozygotic twin pairs reported a negative correlation between fecal SCFAs and adiposity parameters, and individuals with visceral obesity had a lower abundance of *Bacteroides* and *Collinsella* (223). Interestingly, Jiang et al., reported a decrease in *Collinsella* but not in *Bacteroides* in individuals with Graves’ disease (224). In a case-control study, it was found that most patients with metabolic syndrome (37%) had subclinical hypothyroidism (SCH) more frequently than overt hypothyroidism (12%) and overt hyperthyroidism (2%) (225). Currently, most case-control studies have reported an association between obesity and high levels of SCFAs but not gut microbiota richness, at least at the phylum level (226). Additional efforts and studies are needed to understand how the thyroid-gut axis participates in the development of metabolic disorders such as obesity, diabetes, and metabolic syndrome.

### Intestinal dysbiosis in hypothyroidism

An unbalanced microbiota present in the small intestine could have an impact on gastrointestinal function, such as the absorption of key elements for thyroid hormone production or even other nutrients important for fetal growth in pregnant women. Alterations in thyroid gland metabolism caused by hypothyroidism or levothyroxine therapy, as well as diabetes mellitus, are significant contributors to the development of dysbiosis characterized by an increase in bacterial colonization in the small bowel, known as small intestinal bacterial overgrowth syndrome (SIBO), a condition with symptoms such as abdominal discomfort and pain, diarrhea, and weight loss, primarily detected by the lactulose hydrogen breath test (LHBT) where lactulose is a disaccharide that reaches the colon before its absorption, or by the glucose hydrogen breath test (GHBT), where glucose is a monosaccharide absorbed in the first portion of the small intestine (227, 228). These tests have been used to detect SIBO in patients with overt hypothyroidism, revealing an increased incidence of abdominal distention, and treatment of this condition showed improved gastrointestinal symptoms (229). Positive LHBT and GHBT test results were found in pregnant women with subclinical hypothyroidism, a common condition during pregnancy. These patients showed increased levels of TPOAb compared to patients without SIBO; however, it is still unclear whether the dysfunctional flora and its metabolites play a role in inflammatory and immune pathways (228). The relationship between thyroid hormones and intestinal motility is important because it could lead to dysbiosis, as it has been observed in hypothyroidism and its association with altered gastrointestinal motility, which increases the risk of developing SIBO (230).

SCFAs are important for maintaining the health status of the host; however, new studies related to the gut thyroid axis have shown interest in the composition of the intestinal microbiota, and the effect of the products of these metabolites on thyroid disorders remains unknown. Because of the direct or indirect effects of the gut microbiota and its metabolites, they should be considered in the pathogenesis of thyroid disorders (231). In patients with subclinical hypothyroidism, receiving different doses of L-thyroxine showed variations in the relative abundance of certain species from genera with hydrolytic activities, such as the hydrolysis of glucoronate and sulfated iodothyronines like *Odoribacter*, and *Alistipes* (203). The treatment prescribed for this condition is based on hormone replacement, and at least 3.8% of patients are chronic levothyroxine users (232). As the gastrointestinal system is crucial for the absorption of levothyroxine (233), little is known about the effect of these chronic treatments on the intestinal microbiota of patients (232). In addition, the microbiota of women with subclinical and overt hypothyroidism has been evaluated, and both studies found an altered abundance of *Prevotella* genera (234–236), and altered abundances of other commensals and opportunistic pathogens have also been observed in metabolic diseases **(** **Table 1** **)**. In hypothyroid Hashimoto’s thyroiditis, is hypothesized that intestinal microbes play a role in triggering the pathogenesis of this autoimmune thyroid disorder (255, 256). Various studies have shown marked dysbiosis in these patients, with reduced bacterial richness and diversity (257), for example, Ishaq et al. found a reduced abundance of *Prevotella* and *Dialister*, with an important overgrowth of *Bacteroides*, *Escherichia-Shigella* and *Parasutterella* genera, with the increased prevalence of opportunistic gut bacteria *Bacteroides* and other species in HT patients (255). Su et al. found a decreased abundance of *Prevotella* and *Fecalibacterium*, *Bacteroides*, and *Lachnoclostridium* genera, which were enriched in healthy patients, indicating that the composition of the microbiome could be related to the clinical parameters of these patients (256). Animal studies suggest that the microbiota from hypothyroidism patients can cause changes in the thyroid function of mice, which could be related to the reduced abundance of SCFAs-producing bacteria (169). The microbial metabolic processes (energy, carbohydrate, and amino acid metabolism) performed by beneficial microorganisms in patients with HT are affected by the reduced relative abundance of commensals (258), and it is unknown how the levels of SCFAs are in these patients. Where female patients tend to show a different dysbiosis certain microorganisms were more abundant than in male patients, indicating that gender is a factor that should be considered in the dysbiosis found in thyroid diseases (258) **(** **Table 2** **).**

**Table 1:** Microbial changes related to subclinical and overt hypothyroidism, and the relation of the altered microbiota to metabolic diseases.

Thyroid disorder (Ref)	Method of evaluation	Changes in gut microbiota compared to control	Microbial functions	Microorganism also related to metabolic diseases	References
Subclinical Hypothyroidism (203)	Male/Female stools, 16S rDNA V3-V4 regions sequenced with Illumina Hiseq PE250 platform	Increased relative abundance of the genera *Odoribacter* and *Enterococcus* in high and middle dosage of L-thyroxine.	-Starch and glucose metabolism, production of isobutyric and isovaleric acid: Some *Odoribacter* species -Production of acetate: *Enterococcus* species -Potential opportunistic pathogens. Genera *Odoribacter*, and *Enterococcus.*	-Individuals with Hypercholesterolemia ­*Odoribacter.*	(237, 238)
Subclinical hypothyroidism in pregnant women (236)	Pregnant women stools, 16S rRNA V3-V4 regions sequenced with Illumina NovaSeq platform.	Subclinical Hypothyroidism pregnant women (20-23^+6^ weeks) * TPOAb^-^ patients: Increased abundance of ­*Prevotella* * TPOAb^+^ LT_4_^-^ patients: Increased abundance of ­*Prevotella* and reduced *Gammaproteobacteria, Enterobacteriaceae* * TPOAb^+^ ${\text{LT}}_{4}^{+}$ patients: Reduced abundance of *Bacteroidia* and *Prevotella.* Subclinical Hypothyroidism pregnant (28-33^+6^ weeks) * TPOAb^-^ patients: Increased abundance of ­*Blautia* and *Agathobacter* genera. Reduced abundance of *Dorea formicigenerans and Bifidobacterium longum* * TPOAb^+^ LT_4_^-^ patients: Increased abundance of ­*Blautia*, and *Agathobacter.* Reduced abundance of *Actinobacteriota, Coriobacteriia, Actinobacteria, Bifidobacterium, Dorea formicigenerans, and Bifidobacterium longum* * TPOAb^+^ LT_4_^+^ patients: Increased abundance of ­*Blautia, Agathobacter, Streptococcus salivarius*, and *Bifidobacterium longum.*	-High fiber utilizing capacity compared to *Bacteroides. Prevotella.* *-*Propionate production: *Bacteroides, and B. longun.* -Acetate production: Some *Blautia* species -Butyrate production: *Agathobacter.*	-Related to T1D: ¯*Bifidobacterium* ­ *Streptococcus* -Related to T2D: ­*Prevotella* -Related to insulin resistance ­*Prevotella* -Related to protective function against *Bacteroides-*induced glucose intolerance: ­*Prevotella* -Related to visceral fat accumulation: ­*Blautia* -Inflammatory and metabolic regulation: ­*Streptococcus salivariu*s.	(18, 239–248)
Hypothyroidism in pregnant women (235)	Pregnant women saliva and stools, 16S rRNA V3-V4 regions sequenced with Illumina Hiseq 2500.	Oral microbiota Increased ­*Gammaproteobacteria* class*, Streptococcus, Neisseria, Prevotella and Pasteurellaceae.* Intestinal microbiota Increased abundance of ­*Roseburia*, *Pasteurellales, Lachnospira, Prevotella*, and *Parabacteroides.*	-Butyrate production: *Roseburia* *-*Acetate production: *Prevotella*	*-*Related to Weight gain: ­*Gammaproteobacteria, Pasteurellaceae in the oral cavity and Porphyromonadaceae* *in the intestine.* *-*Related to non-alcoholic fatty liver disease: ­*Gammaproteobacteria and prevotella* - Related to increased body mass index ­*Prevotella* in saliva.	(249–252)
Hypothyroidism in pregnant women (234)	Pregnant women stools, 16S rRNA V3-V4 regions, sequenced with Illumina MiSeq platform, and lipid profile determination by LC-MS.	Increased abundance of ­*Prevotella and Haemophilus* Reduced abundance of *Blautia*	-Acetate production: Some *Blautia* species	-Related to plasmatic levels of phosphatidylcholine and sphingomyelin ¯*Blautia* -Might participate in promotion of chronic inflammation: ­*Prevotella strains.* -Some strains related to high fecal SCFAs, obesity and cardiometabolic risk: ­*Haemophilus*	(131, 253, 254)

**Table 2:** Microbial changes related to Hashimoto’s Thyroiditis, and the relation of the altered microbiota to metabolic diseases.

Thyroid disorder (Ref)	Method of evaluation	Changes in gut microbiota compared to control	Microbial functions	Microorganism also related to metabolic diseases	References
Hashimoto’s Thyroiditis (257)	Female stools, 16S rDNAV4 region, sequenced with Illumina HiSeq platform.	Changes in HT patients with normal thyroid function: Increased abundance of ­*Lachnospiraceae incertae sedis, Lactonifactor, Alistipes, and Subdoligranulum* Changes in HT patients with abnormal thyroid function ­Increased abundance of *Bacteroidetes* ­*Phascolarctobacterium and prevotella*	-Butyrate producers: *Lachnospiraceae incertae sedis, Subdoligranulum* -Acetate and propionate producer: *Phascolarctobacte-rium* species.	-Considered pathobiont promoting chronic inflammation: Some *Prevotella* species. -Related to T2D: ­*Subdoligranulum* -Related to metabolic syndrome: ­*Bacteroidetes* -Related to heart rate variability (HRV) ­*Lachnospiraceae* species.	(253, 259–263)
Hashimoto’s Thyroiditis (258)	Male and Female stools, 16S rDNA V3-V4 regions, sequenced with Illumina Miseq PE300 platform	Changes in HT patients with normal thyroid function Increased abundance of ­ *Lachnoclostridium*, *Holdemaniawere, Akkermansia, Ralstonia, Fournierella*, and *Megamonas* Changes in HT patients with abnormal thyroid function Increased abundance of ­*Akkermansia*, *Acetitomaculum*, *Shuttleworthia*, *Flavobacteriaceae, Lachnospiraceae family, Oscillospirales* Reduced *Klebsiella* Changes in Female HT patients Reduced *Bifidobacterium, Klebsiella* ­Increased *Megamonas* Changes in Male HT patients Increased ­ *Bifidobacterium*	-Propionate production from mucin degradation: *Akkermansia muciniphila*	-Related to T1D: ¯ *Bifidobacterium* -Related to T2D: ­*Lachnospiraceae* family, *Megamonas.*	(246, 259, 264, 265)
Hashimoto’s Thyroiditis (255)	PCR-DGGE of 16S rRNA V3 region, and 16S rRNA V4 region pyrosequencing	Increased ­Abundance of *Bacteroides*, *Escherichia-Shigella* and *Parasutterella* genera. Reduced abundance of *Prevotella_9*, *Dialister, Bifidobacterium* and *Lactobacillus genera.* Increased ­ Prevalence of opportunistic bacteria *Bacteroides uniformis, Bacteroides pyogenes, Bacteroides vulgates, Shigella dysenteriae, Bacteroides intestinalis, Escherichia coli, Sporomusa ovate, Bacillus* sp.*, Shigella flexneri.*	-Dietary fiber fermenter: *Prevotella* and *Bacteroides*.	-Related to insulin resistance: *Bacteroides vulgates* -Related to T1D: ¯ *Bifidobacterium, Bacteroides* species. -Related to T2D: *B. vulgates*, *B. intestinalis*, *Escherichia.* -Related to cushing’s syndrome: ­ *Escherichia-Shigella* -Related to metabolic syndrome: *Prevotella_9*	(244, 245, 259, 265–268)
Hashimoto’s Thyroiditis (256)	Male/Female stools, 16S rRNA V3-V4 regions, sequenced with Illumina Hiseq 2500 platform.	Increased abundance of ­ratio Firmicutes/Bacteroidetes (F/B) Increased abundance of ­Lachnospiraceae Family Increased abundance of ­*Blautia, Dorea, Roseburia*, *Butyricicoccus, Streptococcus, Fusicatenibacter, Coprococcus_2, Subdoligranulum, Romboutsia, Anaerostipes*, *Ruminococcus_gauvreauii_group, Ruminococcus_torques_group, Fusicatenibacter* and *Eubacterium_hallii_group* genera. Reduced abundance of *Fecalibacterium, Bacteroides, Prevotella_9* and *Lachnoclostridium* genera	-Butyrate producers: *Eubacterium hallii, Anaerostipes.* -Acetate producers: *Blautia*	-Related to metabolic syndrome: ­*Bacteroidetes, Prevotella_9.* -Related to T1D: ­*Streptococcus.*	(133, 242, 261, 267, 269–271)

### Intestinal dysbiosis in hyperthyroidism

In patients with Graves’ disease (GD), an autoimmune disease of the thyroid gland, a clearly altered composition of the gut microbiota has been described (272). At the phylum level, GD patients had a significantly lower proportion of *Firmicutes* and a significantly higher proportion of *Bacteroidetes *compared with the controls. This indicates that thyroid hormones may also affect the composition and function of the microbiota, which in turn leads to changes in a person’s weight (224). At the genus level, GD patients had an altered abundance of *Faecalibacterium*, *Bacteroides*, *Prevotella*, and *Bifidobacterium* and lower numbers of *Blautia, Subdoligranulum*, *Eubacterium species*, compared to controls. The functional prediction has revealed that *Blautia* may be an important microbe in certain metabolic pathways that occur in the hyperthyroid state (224). Another study showed an altered and significantly decreased abundance of metabolic abilities in fatty acid biosynthesis, creatinine degradation, pyruvate fermentation to hexanol, anaerobic energy metabolism, and gluconeogenesis (128). These patients with GD showed reduced levels of SCFAs (127, 128), which could be related to the susceptibility of these patients to intestinal epithelial cell injury and increased intestinal permeability by alterations in the tight junctions in the intestinal epithelial cells, according to studies in patients with GD who had significantly higher serum levels of serum LPS, intestinal fatty acid-binding protein (I-FABP), zonulin, and D-lactate (273).

Other authors have related the increased abundance of *Bacteroides* to its ability to produce SCFAs other than butyric acid, including succinate, propionate, and acetate, indicating that it might be implicated in the impairment of intestinal barrier function because these metabolites do not induce mucin production (224), resulting in the release of a large number of pro-inflammatory factors outside the intestine and causing immune dysfunction (224). A study evaluating the combination of the treatment for GD, methimazole (MI) in combination with potential prebiotic berberine supplementation, showed an improved abundance of beneficial species like *Lactococcus lactis* while decreasing the abundance of pathogenic bacteria like *Enterobacter hormaechei* and *Chryseobacterium indologenes*, and also improved FT_3_ and TSH indices to normal levels, relating TSH to *Faecalibacterium prausnitzii* and enterobactin biosynthesis, and with *Lactococcus lactis* were positively correlated with the three metabolic pathways of vitamin K2 synthesis, whereas MI alone restored only FT3 and had no modifications on the microbiota (274), demonstrating the importance of evaluating the combination of treatments that could provide benefits for the host microbiota and how to ameliorate the symptoms of the disease. Other studies found an increase in pathogenic bacteria and opportunistic pathogens in these patients, which are related to significant differences in the reduced abundance of acetic, propionic, and butyric acids with an increased abundance of cholate and chenodeoxycholate (128). Even when these metabolites were detected *in silico* (275), their identification is considered a potential biomarker for the disease. In both autoimmune thyroid diseases (AITDs), besides showing differences in the core microbioma of GD and HT patients, in both were found concrete bacteria that could be related to their reduced tolerance to self-antigens (276) (**Figure 3**). Therefore, evidence suggests a key role of microbiota status in thyroid diseases, where metabolic changes and dysbiosis are related to an altered composition of SCFAs producers and an increased abundance of pathogens **(** **Table 3** **)**.

**Table 3:** Microbial changes related to Hyperthyroidism and Grave’s Disease, and the relation of the altered microbiota to metabolic diseases.

Thyroid disorder (Ref)	Method of evaluation	Changes in gut microbiota compared to control	Microbial functions	Microorganism also related to metabolic diseases	References
Hyperthyroid patients (277)	PCR-DGGE of 16S rRNA V3 region.	Reduced abundance of *Bifidobacterium* and *Lactobacillus* Increased abundance of ­*Enterococcus, Clostridium*	-Production of SCFAs: *Lactobacillus.*	-Related to T1D: *Bifidobacterium, Clostridium.* -Related to T2D: ­*Clostridium.* -Related to improvement of inflammatory disorders: *Lactobacillus* species.	(259, 269, 270, 278)
Hashimoto’s Thyroiditis & Graves’ Disease (276)	16S rRNA of V2-4-8 and V3-6, 7-9 regions, sequenced with Ion Chef System and Torrent S5TM System	Grave’s Disease patients: Reduced abundance of *Rikenellaceae* Family Increased abundance of ­ *Prevotellaceae* ­*Fusobacterium, Sutterella.* Reduced abundance of *Faecalibacterium, Rikenellaceae*	-Butyrate producer: *Fusobacterium*	-Related to obesity: ­*Fusobacterium*	(279, 280)
Graves’ Disease (272)	PCR-EDGE of 16S rRNA of V3 region, RT-PCR and 16S rRNA V3-V4 regions, sequenced with Illumina Hiseq 2500 platform.	Increased ­Proportion of Bacteroidetes, Actinobacteria, and Proteobacteria Phyla. Increased ­ Abundance of *Prevotella_9* and *Haemophilus* genera. ­ *Bacteroides vulgatus* Reduced Abundance of *Alistipes* and *Faecalibacterium* genera. Reduced *Lactobacillus* and *Bifidobacterium* genus Reduced *Clostridium leptum*	-Butyrate production: Proteobacteria	-Related to obesity: ­Bacteroidetes, Proteobacteria Phyla ¯ *Clostridium Alistipes, Bifidobacterium, Lactobacillus* *leptum.* -Related to metabolic syndrome: ­Bacteroidetes, *Prevotella_9* -Related to T1D: ­ *Bacteroides vulgatus*	(259, 261, 270, 281–283)
Graves’ Disease (224)	16S rRNA V3-V4 regions, sequenced with Illumina MiSeq PE300 platform	Increased ­Proportion of Bacteroidetes phyla Reduced Proportion of Firmicutes phyla Increased abundance of ­*Bacteroides* and *Lactobacillus* genus. Reduced abundance of *Blautia*, *Eubacterium hallii* group, *Anaerostipes*, *Collinsella*, *Dorea*, unclassified *Peptostreptococcaceae*, and *Ruminococcus torques* group genus.	-Acetate producer*: Blautia* -Butyrate producer: *Anaerostipes*	-Related to insulin resistance: *Peptostreptococcaceae* -Related to T1D: ¯ *Blautia*, -Related to T2D: *Peptostreptococcaceae* -Related to metabolic syndrome: *Peptostreptococcaceae*	(242, 246, 261, 269, 281, 284)
Grave’s Disease (128)	Male/Female stools sequenced with Illumina HiSeq 2500 platform and SCFAs determination by GC-MS	Increased abundance of ­*Coprobacillus*, *Streptococcus* and *Rothia* genera ­*Eggerthella lenta*, *Streptococcus parasanguinis, Veillonella parvula, Fuso-bacterium mortiferum and Streptococcus salivarius.* Reduced abundance of *Faecalibacterium prausnitzii, Butyricimonas faecalis, Bifidobacterium adolescentis* and *Akkermansia muciniphila*	-Propionate production from mucin degradation: *Akkermansia muciniphila* -Propionate production: *Veillonella parvula* -Butyrate production: *Faecalibacterium prausnitzii, Bifidobacterium adolescentis*	-Related to T1D: ­*Streptococcus* -Related to T2D: ­ *Eggerthella lenta, Akkermansia muciniphila* *-*Ameliorate T2D: *Bifidobacterium adolescentis.*	(139, 264, 285–287)
Grave’s Disease Patients (127)	Male/Female stools, 16S rRNA V1-V2 regions, sequenced with Illumina HiSeq 2500 platform, and SCFAs determination by GC-MS.	Increased abundance of *Bacteroides, and Yersinia enterocolitica.* SCFAs reduced in GD. ¯ Propionic and butyric acid.	-Propionic acid producer: *B. fragilis.*	-Glucose intolerance: *Bacteroides* -Associated to blood lipid levels: *Turicibacter.*	(237, 239, 243, 280)
Grave’s Disease and improved therapy with Prebiotic (274)	Stools from baseline, 3 month and 6 months after treatment, sequenced with Illumina HiSeq 2500 platform.	Patients treated with Methimazole: Reduced abundance of *Prevotella* spp, *Streptococcus* *pneumoniae, Selenomonas ruminantium, and Enterobacter hormaechei.* Patients treated with Methimazole and potential prebiotic Berberine: Increased abundance of ­ *Lactococcus lactis, Enterococcus hirae* Reduced abundance of *Prevotella* spp., *Chryseobacterium indoltheticum* and *Tannerella forsythia*. *Faecalibacterium prausnitzii* and *Lactococcus lactis* were positively correlated with TSH levels.	-Butyrate producer: *Faecalibacterium prausnitzi, Enterobacter hormaechei* -Propionate, acetate production: *Selenomonas ruminantium*	-Related to T2D: ¯*Faecalibacterium prausnitzi*,	(254, 259, 285, 288)

## SCFAs and Thyroid function

There is limited information available of the thyroid gut-axis besides knowing the dysbiosis found in thyroid disorders as it was described in the previous section, highlighting new findings related to the content of SCFA of these patients. Nevertheless, little is known about how SCFA affect thyroid function or how they cooperate (289). However, it is important to revisit that a primary contribution of SCFA’s to cell homeostasis is the regulation of HDAC (251), especially butyrate and propionate can inhibit HDAC (184), preventing the removal of acetyl groups on certain lysine residues in histones and non-histone proteins that control gene expression (188). Other described epigenetically active mechanisms used by SCFAs are histone butyrylation (by the conversion of butyrate to butyryl-CoA) or propionylation (by the converting propionate to propionyl-CoA) (290). Histone acetylation may be crucial for the control of hormone-mediated transcriptional regulation (291), and some studies have shown that butyrate can regulate thyroid hormone receptors levels through the acetylation of chromatin-associated proteins in rat cells (292–294), promoting the expression of the growth hormone (GH) which is regulated by T_3_ and retinoic acid receptors, as result of the accumulation of hyperacetylated histones (291). Moreover, it has been described that the treatment of these animals with n-butyrate, could modulate gene expression by increasing the binding capacity of T_3_ on nuclear thyroid hormone receptors in the rat liver (295).

Even when the *in vivo* and *in vitro* studies about the evaluation of thyroid hormone receptors and butyrate here mentioned are at least 40 years old. It is important that new studies of the gut-thyroid axis, could generate new information that laid the foundations for the role of the microbiota and its metabolites in thyroid function, which is quite useful for developing potential treatments for thyroid-associated diseases (296), especially considering that autoimmune diseases like HT have increased inflammation that could be related to dysbiosis that leads to altered levels of SCFAs (297), and the bacterial metabolites have an important role in maintaining a balance the pro- and anti-inflammatory environment (184).

## Methods and insights on the detection of SCFAs

### Technologies and approaches in the detection of SCFAs

Given the great importance of SCFAs as bacterial metabolites produced by components of the microbiota and their relevance in processes including regulation of metabolism, immune system development and activation, improving gut barrier function, and their role in the occurrence of metabolic diseases such as thyroid diseases (132), it is essential to have one or more robust methods to measure the levels of these molecules in biological samples such as feces, luminal contents, or serum. Evidence has suggested that for detecting SCFAs, it is necessary to perform a meticulous analysis of these SCFAs with an accurate extraction and sample preparation process to guarantee the correct SCFAs profiling (298, 299). In this context, several methods of SCFAs extraction, purification, and treatment of biological samples have been described, including techniques of sample derivatization and methods of purification to obtain a fast sample preparation (300). The unique physicochemical properties of SCFAs, such as their high polarity, low vapor pressure, high volatility, and complex matrix of biological samples, require highly sensitive, selective, and accurate methods for their determination (299). In this sense, diverse methods have been developed to detect SCFAs in biological samples (300), including separation-based techniques such as gas chromatography (GC), high-performance liquid chromatography (HPLC), or mass spectrometry (MS), and their hyphenation with MS (GC/MS or HPLC/MS) the most common methods (301–305). Nevertheless, techniques such as capillary electrophoresis (CE) or nuclear magnetic resonance (NMR) are frequently used (306, 307). Within the analytical methods for SCFAs, the GC remains the most commonly used technique because of its high resolution and sensitivity (299), in addition to the advantage of being able to be attached to a flame ionization detector (FID) (308, 309) or to a mass spectrometer (MS) (303, 310–312), that can further enhance the selectivity and sensitivity of the method. However, the HPLC technique is also favored for its sensitivity and ability to handle complex samples (313), as well as the advantages of coupling fluorescence, ultraviolet–visible light (UV–VIS) (304), electrochemical detection (ECD) (314) and MS detectors (315, 316). Both methods involve the separation of SCFAs based on their physical and chemical properties, followed by the detection and quantification of these metabolites. Although the GC/MS and HPLC/MS methods offer excellent SCFAs analysis alternatives, they often require a sample chemical derivatization process with specific techniques according to the method to be used, to improve their volatility and/or retention properties in the column of equipment (300). However, it is necessary to carry out an extraction and purification process before SCFAs derivatization and analysis because for some samples the detection of small concentrations of SCFAs can be challenging due to high concentration of other biological constituents, which complicates the precision and reproducibility of the method and results in instrumental contamination, which makes it challenging to analyze and quantify SCFAs. As a result, it is crucial to perform a selective extraction and correct derivatization of SCFAs.

Several physical pretreatments are used to enhance and accelerate SCFAs extraction processes, such as filtration, ultrafiltration, or centrifugation, which are fast and simple, but, despite the rapidity of these extraction methods, have the drawback that a large number of impurities may also be extracted, leading to incomplete separation of the SCFAs and therefore contaminating the chromatographic column reducing its useful life (310, 317, 318). The use of steam distillation and vacuum distillation, which are carried out at low temperatures and pressure, are other methods for the separation of SCFAs, however, over a long period can generate the possibility of a loss of volatile acids (319, 320). One extraction strategy widely used is the acidification of these compounds, which enhances the hydrophobicity and therefore facilitates extraction with apolar organic solvents, which could be complemented with a liquid-liquid extraction (LLE) (321, 322). In this same context, extraction in the absence of solvent employing a solid phase microextraction device (SPME) taking advantage of the volatility of SCFAs is a technique that is increasingly used due to its speed, selectivity, and sensitivity (308, 312).

On the other hand, as previously mentioned, a frequently used approach for the quantification of SCFAs involves chemical derivatization followed by analysis using separation-based techniques. The derivatization strategies allow the change in the SCFAs properties and have been used to enhance the compound’s separations and increased resolution allowing for more accurate and sensitive measurement. The most common techniques for the derivatization of SCFAs are the conversion of SCFAs to their corresponding fatty acid esters or the corresponding methyl esters, derivatives that enhance their volatility and improve detection by gas chromatography (GC) or mass spectrometry (MS) (300, 323). In contrast, converting SCFAs to their corresponding 2,4-dinitrophenylhydrazine derivatives or Trifluoroacetic acid (TFA) treatment are derivatization strategies used to be easily quantified using high-performance liquid chromatography (HPLC) (304, 324). It is important to note that each derivatization technique has its own set of advantages, and the choice of an adequate method will depend on the specific application and the analytical requirements. Finally, another alternative SCFAs determination method is enzymatic detection. This technique involves the use of specific enzymes to convert SCFAs into a detectable product, which can then be quantified using various methods, such as UV spectrophotometry. In addition, the direct detection method for SCFAs is the ELISA technique (Enzyme-Linked Immunosorbent Assay), which utilizes antibodies specific to the SCFAs (325). Although ELISA is a rapid and simple method, however it is less sensitive than GC, HPLC, or MS. It is important to note that the choice of detection method will depend on the specific application, the type of sample, and the desired sensitivity and specificity. Furthermore, the use of derivatization techniques, as discussed earlier, can enhance the detection and analysis of SCFAs. In conclusion, the detection of SCFAs is crucial for understanding their role in various physiological and pathological processes and the development of new therapies. The advancement of analytical techniques, along with the increasing recognition of the importance of SCFAs, will likely continue to drive new insights into their detection and characterization.

### Projections of SCFAs as strategies for following thyroid diseases

Even though SCFAs have been studied in the context of thyroid disorders, there is limited supporting evidence. Gas chromatography-mass spectrometry (GC-MS) measurements of SCFAs in patients with Grave’s disease revealed decreased levels of acetic, propionic, and butyric acid (127, 128). However, more studies in other thyroid disorders are needed, although it has been suggested that SCFAs may indirectly influence thyroid function by affecting gut microbiota and immune function (326). Given that butyrate, which plays a role in regulating the immune system and has anti-inflammatory effects that could be potentially beneficial for people with AITDs such as Hashimoto’s thyroiditis (327).

Although there is ongoing research to identify new biomarkers for thyroid diseases, and SCFAs may be among the compounds studied in this context, more investigation is needed to understand the relationship between SCFAs and thyroid disorders.

## Conclusion

Thyroid disorders are involved for the development of diseases or chronic metabolic conditions such as type 1 diabetes and type 2 diabetes. Iodine nutrition is considered the main cause of thyroid disorders’ development. In this context, adequate nutrition can be a protective factor to avoid the development of thyroid disease and improve thyroid function. Key nutrients like iodine, selenium, and iron uptake, can be affected by an alteration of the gut microbiota homeostasis, influencing the host’s systemic metabolism. Therefore, THs availability and a balanced gut microbiota are key for the regulation of metabolism. Thyroid disorders are more common in women, and the relationship between gut microbiota, thyroid conditions during pregnancy, and their effects on the offspring’s immune system is far from being completely understood. What is known is that dysbiosis is often observed in thyroid disorders, but the enzymatic and metabolic pathways affected by the abundance or depletion of certain microorganisms in these conditions need to be described. Here we show that there is no strong research demonstrating the impact of SCFAs on thyroid function, even though there is no doubt that SCFAs have beneficial effects on important organs, tissues, and cells, considering that SCFAs allowing chemical communication both locally at the intestinal mucosa and systemically to the rest of the organs. In this work, it is described how a low abundance of beneficial organisms correlated with altered SCFAs production, highlighting the importance of SCFAs detection as an evaluation tool for the patient’s gut microbiota’s environment and the need for more research on metabolites linking gut dysbiosis and thyroid function to improve the patient’s life quality.

## Author contributions

Conception, design, illustrations of **Figures 2**, **3**, drafting of the manuscript (MM-L). MM-L, AM, AR, EG-M, MR-R, OA-M, OV, SB, YD and MO wrote sections of the manuscript. CR, MO, AM and FM-G contributed to the literature review and manuscript revision. AK, CM contributed with manuscript revision. All authors contributed to the article and approved the submitted version.
